# Identification of attractive blend for spotted wing drosophila, *Drosophila suzukii*, from apple juice

**DOI:** 10.1007/s10340-018-1006-9

**Published:** 2018-06-22

**Authors:** Yan Feng, Robert Bruton, Alexis Park, Aijun Zhang

**Affiliations:** Invasive Insect Biocontrol and Behavior Laboratory, Agricultural Research Service, United States Department of Agriculture, Bldg. 007, Rm. 312, BARC-W, Beltsville, MD 20705 USA

**Keywords:** Fruit fly, Attractant, Fermentation, Acetoin, Ethyl octanoate, Insect pest management

## Abstract

*Drosophila suzukii*, commonly known as the spotted wing drosophila (SWD), is an exotic fruit fly from Southeast Asia that was introduced to the temperate regions of North America and Europe in 2008. It attacks a wide variety of fruits and has become a devastating pest of soft-skinned fruit crops. Due to the rapid spread of SWD across the newly invaded continents, fresh fruit markets have a zero-tolerance policy regarding *D. suzukii* infestation. Specific and efficient *D. suzukii* detection tools are urgently needed so that farmers can deliver timely management interventions to meet market demands. Since SWD is known to be attracted to damaged and rotting fruits, headspace volatiles from fresh and fermented apple juices were collected and analyzed by gas chromatography–mass spectrometry. Special attention was given to the compounds produced and/or enriched during the fermentation process. After performing a series of laboataory and field tests, we identified a quinary blend, which is more efficient and selective for *D. suzukii* than the currently standard apple cider vinegar and commercially available SWD lure under field conditions. Identification of SWD attractant will help growers accurately detect *D. suzukii* adult infestations in orchards, thereby allowing for timely pest management interventions while reducing conventional insecticidal usage to protect our crops, environment, and ecosystem.

**Electronic supplementary material:**

The online version of this article (10.1007/s10340-018-1006-9) contains supplementary material, which is available to authorized users.

## Key message

- *Drosophila suzukii* attacks a wide variety of fruits and has become a devastating pest of soft-skinned fruit crops.
- Early detection of this fruit fly on farms is essential for quick management measures that could lead to reductions in the rate and amount of insecticide applications.
- A quinary blend was identified from the headspace volatiles of apple juice.
- This blend is more efficient and selective for attraction of *D. suzukii* than the currently standard apple cider vinegar (ACV) under field conditions.

## Introduction

The spotted wing drosophila (SWD), *Drosophila suzukii* Matsumura (Diptera: Drosophilidae), is an invasive fruit-infesting fly native to Southeast Asia (Calabria et al. 2010). Since it was accidently introduced to central California in August 2008, *D. suzukii* has rapidly spread across the USA (Hauser 2011). It has also been detected in Canada, Mexico, and Europe (Asplen et al. 2015; Lee et al. 2011). *Drosophila suzukii* attacks a wide variety of fruits and has become a devastating pest of soft-skinned fruit crops such as raspberries, blueberries, cherries, and strawberries (Beers et al. 2011; Hauser 2011). Unlike most drosophilid flies that feed and oviposit on overripe, damaged, or decomposing fruits, *D. suzukii* can feed and oviposit on sound ripening fruits (Calabria et al. 2010; Hamby et al. 2016; Mitsui et al. 2006). The females possess a serrated ovipositor, which allows them to cut through the epicarp of their hosts. Fruit infestation by *D. suzukii* larvae has resulted in significant financial losses to farmers (Cini et al. 2012; Walsh et al. 2011).

Due to its economic impact on fruit crops (Goodhue et al. 2011), farmers usually resort to calendar-based applications of insecticides to manage *D. suzukii* (Beers et al. 2011; Lee et al. 2011). Detection of this fly on farms is essential for quick management measures, which will reduce the rate and amount of insecticide applications required to avoid economic loss. Since *Drosophila* spp. are strongly attracted to overripe, fallen, rotting fruit (Mallis 1969), traps baited with fermentation products such as apple cider vinegar (ACV), wine, or yeast as baits are employed for SWD populations monitoring on farms (Beers et al. 2011; Hamby and Becher 2016; Landolt et al. 2012b; Lee et al. 2012). In particular, ACV is commonly used because it is easily available and relatively cheap (Beers et al. 2011; Lee et al. 2011); however, ACV is not adequately selective and effective for SWD detection (Tonina et al. 2018). In studies testing different trap designs using ACV as bait, it was found that only 26–31% of the total numbers of captured *Drosophila* were *D. suzukii* (Lee et al. 2012) and high numbers of two beneficial parasitoids were found in the traps (Wang et al. 2016). In addition, ACV-baited traps are ineffective at detecting flies before populations reach an economic injury level leaving farmers without sufficient time to apply protective treatments (Burrack et al. 2015; Lee et al. 2012). Therefore, many researches focused on identification of the key attractive compounds for *D. suzukii* from wine, yeast, and SWD preferred fruits have been carried out. Although 11-component blend based on raspberry volatile (Abraham et al. 2015) and eight-component (Cha et al. 2012) and four-component (Cha et al. 2014, 2015) blends based on wine and yeast volatiles and some commercial synthetic lures have been developed and tested as SWD attractants, they are unsatisfactory for the demands of SWD adult infestation detection and population monitoring because of their poor selectivity and efficiency (Basoalto et al. 2013; Burrack et al. 2015; Cha et al. 2013; Iglesias et al. 2014; Kirkpatrick et al. 2017; Kleiber et al. 2014; Mazzetto et al. 2015; Tonina et al. 2018). Current monitoring systems suffer from inconsistent efficacy, and they exhibit variability in trap captures depending on crop type, crop phenology, and *D. suzukii* phenology and are difficult to relate trap captures to infestation (Hamby and Becher 2016). More effective and selective attractants are needed for detecting, monitoring, and managing this invasive species.

This work focuses on developing more effective and selective attractants for *D. suzukii* based on volatiles from fruit aromas. Since the yeast microbes volatiles from ripening fruits could induce strong attraction in *Drosophila* larvae and flies (Becher et al. 2010, 2012; Hamby and Becher 2016), headspace volatiles collected from fresh and fermented apple juices were compared. Special attention was given to the compounds produced and/or enriched during the fermentation process. This newly identified quinary chemical blend is expected to lure SWD flies more efficiently and selectively into traps in the early season and attract them to insecticide strips or biocontrol dispensers. It will be vital for SWD adult infestation detection and population monitoring in support of SWD management programs. The new blend will provide opportunity to enable future development of mass trapping and attract-and-kill technologies for control of this exotic pest.

## Materials and methods

### Apple juice headspace volatile collections and raspberry solvent extract preparation

The headspace of apple juice was collected using fresh and fermented Old Orchard^®^ brand apple juice (100% apple juice from concentrate with vitamin C; no sugar, artificial flavors, colors, or preservatives added; Shoppers Food and Pharmacy, College Park, MD) in August 2013. Fresh apple juice (250 mL, obtained from store and used right away) was introduced into a 1-L four-necked glass container (Zhang et al. 1994, 1999). Air was drawn into the container through 6–14-mesh activated charcoal (Fisher Scientific, Pittsburgh, PA) and out of the container through two traps (15 cm × 1.5 cm o.d.) containing Super Q (200 mg each; Alltech Associates, Inc., Deerfield, IL) by vacuum (~ 1 L/min). For fresh apple juice volatile collection, aeration was conducted for 3 h at room temperature in daytime. Adsorbents were eluted with methylene chloride (4 × 0.5 mL); the elutes (2 mL/each sample) were concentrated to ~ 500 μl under a nitrogen stream and stored at − 30 °C for future gas chromatography (GC)–mass spectrometry (MS) analysis. For fermented apple juice volatile collection, the same container was set on the laboratory benchtop for 20 days while the apple juice fermentation occurred inside (ACV changed into unclear and turbid solution). Aeration was then conducted for 3 h, and adsorbent elution was carried out in the same manner as fresh apple juice volatile. A polydimethylsiloxane-coated solid-phase microextraction (SPME) fiber (PDMS. 100 µm, Supelco Inc., Bellefonte, Pennsylvania) was used for apple juice SPME sampling (Zhang et al. 1999). Raspberry extract was prepared at Rutgers University from fresh raspberry fruit by homogenized blending and centrifuging (Abraham et al. 2015). The resulting extracts containing water-soluble organic substances were stored in a freezer (− 10 °C) for laboratory bioassay (Abraham et al. 2015).

### GC and GC–MS spectrometry

The GC and electronic impact (EI) GC–MS systems used were as described (Zhang et al. 2004). A Hewlett Packard (HP) 6890 GC was coupled to a flame ionization detector (FID) using DB-WAXETR or DB-5 capillary column (60 m × 0.25 mm i.d., 0.25-μm film thickness, J&W Scientific Inc., Folsom, CA). Oven temperature was started at 50 °C for 2 min, then programmed to rise to 230 °C at 15 °C/min, and held for 20 min in the splitless mode with hydrogen as carrier (2 mL/min). For GC–MS analysis, a HP 6890 GC was coupled to a HP 5973 Mass Selective Detector (MSD) using the same columns as GC-FID, but with helium as carrier (1.4 mL/min). A 70 eV electron beam was employed for sample ionization. The chemical identification of the headspace volatiles was based on comparison of their mass spectra with the NIST and Wiley mass spectral libraries, and identities were confirmed by mass spectra and GC retention times of authentic standards on both polar and nonpolar GC capillary columns (Zhang et al. 1999). The ratios of identified components were determined by using GC–FID.

### Insect rearing

Laboratory-reared *D. suzukii* flies were obtained from Rutgers University in 2014 with permission (permit: P526P-14-00,267) from the Animal and Plant Health Inspection Service (APHIS). The original colony was established in July 2012 from *D. suzukii*-infested high-bush blueberry Bluecrop cv. (*Vaccininum corymbosum* L.) fruits in Burlington County, New Jersey (Abraham et al. 2015). Insects were reared on cornmeal diet (Dalton et al. 2011) in polystyrene tubes (height 95 mm, diameter 28.5 mm, Fisher Scientific, PA, USA) with ventilated plugs (height 25 mm, diameter 28.5 mm, Fisher Scientific, PA, USA). The colony was maintained in an incubator under ~ 25 °C, 60% RH, and a 16:8-h (L/D) photoperiod. Maintenance of the *D. suzukii* colony was achieved by transferring emerging adults into new diet tubes on a weekly basis. All *D. suzukii* used in bioassays (2–8 days post-eclosion) were counted and sexed based on the presence of a dark spot on the wing tips of males and the presence of a serrated ovipositor in females (Walsh et al. 2011) for overall colony sex ratio determination. The sex ratio of laboratory-reared *D. suzukii* was determined to be close to 1:1.

### Chemicals

All chemicals used in this work were purchased from Sigma-Aldrich (St. Louis, MO, USA), including isobutanol (IB), 99 ≥ %, CAS 78-83-1; 2-methyl-1-butanol (2 MB), 99 ≥  %, CAS 137-32-6; 3-methyl-1-butanol (3 MB), 98.5 ≥  %, CAS 123-51-3; ethyl hexanoate (EH), 99 ≥  %, CAS 123-66-0; acetoin (AT), 99%, CAS 512-86-0; ethyl octanoate (EO), 99 ≥  %, CAS 106-32-1; acetic acid (AA), 99.7 ≥  %, CAS 64-19-7; benzaldehyde (BA), 98 ≥  %, CAS 100-52-7; ethyl decanoate (ED), 99 ≥  %, CAS 110-38-3; methyl benzoate (MB), 99%, CAS 93-58-3; phenethyl alcohol (PE), 99 ≥  %, CAS 60-12-8; as well as ethanol (EtOH), 200 proof HPLC grade, ethyl acetate (EA), methylene chloride, and pentane, HPLC grade. They were used without further purification. Pentane was used as a solvent for dilution when lower-than-stock concentrations of any of the aforementioned chemicals were required.

### Field tests

Commercially available Victor^®^ Yellowjacket & Flying Insect Traps (Great Lakes IPM Inc., Vestaburg, MI) filled with ~ 300 mL of tap water containing a surfactant (Seventh Generation™ Natural Dish Liquid—Free & Clear, Shoppers Food and Pharmacy, College Park, MD, 4 mL/gallon) as a drowning solution were used in all field tests at the Beltsville Agricultural Research Center-West, Beltsville, MD (10300 Baltimore Ave., Beltsville, MD) and at the blueberry field at Butler’s Orchard (22222 Davis Mill Rd, Germantown, MD). No pesticide was sprayed during the trapping experiments. Unless otherwise indicated, pure chemical (individual or blend, 1 mL) was loaded onto a cotton ball held within a polypropylene flex micro-centrifuge tube (1.5-mL Eppendorf micro-centrifuge tube, VWR International, Radnor, PA), and the lid of the tube was then closed. Tubes filled only with a cotton ball were used as blank controls. The micro-centrifuge tube lure was opened (S1 Fig.), and the lid was snapped into one of four holes on the cap of the Victor^®^ trap, leaving the three remaining holes to serve as entrances for attracted insects (S2 and S3 Fig.). At the Beltsville Agricultural Research Center, traps were hung from the branches of trees (S4 Fig.) on the edge of a small woodlot in blocks consisting of 3–5 treatments and one blank control with the traps spaced approximately 5 m apart within a group. Three to 15 replications were tested for each treatment (for ethanol effect: *N* = 10, Fig. 4a, and acetic acid effect: *N* = 15, Fig. 4b, during October 2015; for ethyl octanoate effect: *N* = 3, Fig. 4c, during November 2015; for ethyl acetate effect: *N* = 3, Fig. 4d, during November 2016; for comparison with Scentry commercial lure: *N* = 6, Fig. 5a, b, during November and December 2016; for phenethyl alcohol effect: *N* = 6, Fig. 5c, during December 2016). The treatments were randomly arranged at different positions weekly within the block, and blocks were separated by 10–20 m. At the blueberry field in Butler’s Orchard, traps were hung from the branches of blueberry bushes, deployed in blocks consisting of different treatments with the traps spaced approximately 5 m apart in a group. Nine to 12 replications were tested for each treatment (for a quinary blend test: *N* = 9, Fig. 5d, during December 2016; for comparison with ChemTica commercial lure: *N* = 12, Table 3, during July 2017). The traps were randomly arranged at different positions within the block, and blocks were separated by 10–20 m.

**Fig. 1:**
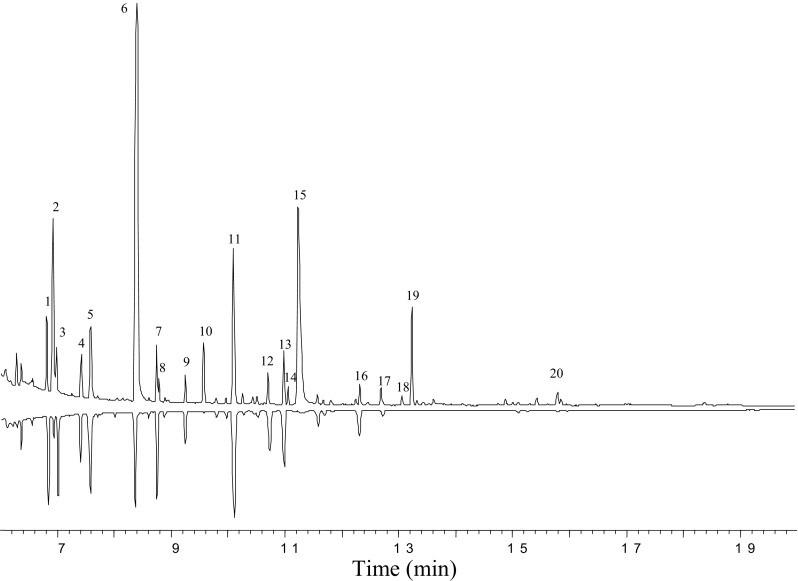
GC–MS traces (total ion) of apple juice volatile extracts of fermented (top) versus fresh (bottom) on DB-Waxetr capillary column. **1**. Butyl acetate, **2**. isobutanol, **3**. hexanal, **4**. 2-methylbutyl acetate, **5**. butanol, **6**. 2- and 3-methyl butanol, **7**. *trans*-2-hexenal, **8**. ethyl hexanoate, **9**. hexyl acetate, **10**. 3-hydroxy-2-butanone (acetoin), **11**. hexanol, **12**. *trans*-2-hexenol, **13**. unknown. **14**. ethyl octanoate, **15**. acetic acid, **16**. benzaldehyde, **17**. dimethyl sulfoxide, **18**. ethyl decanoate, **19**. methyl benzoate, **20**. phenethyl alcohol

**Fig. 2:**
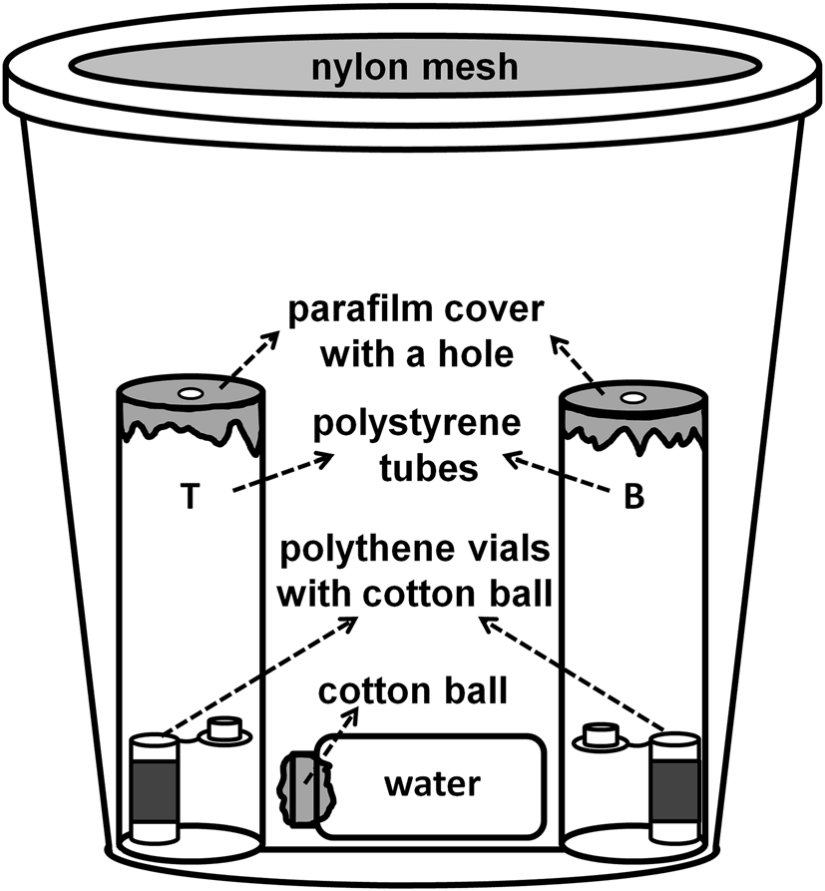
Laboratory dual-choice bioassay apparatus used in experiment. *T* treatment tube, *B* blank control tube

The preliminary field tests were conducted at the Beltsville Agricultural Research Center during October 17–24, 2014, using six different lures: (1) apple juice blend [treatment 1, seven components based on apple juice fermentation products (Fig. 1)], (2) raspberry blend (EAG active 11-component blend) (Abraham et al. 2015), (3) single component [2-methyl butanol (2 MB)], (4) [3-methyl butanol (3 MB)], (5) binary components blend [(2 MB + 3 MB in 1:1 ratio)], and (6) blank control (Table 1, *N* = 3). Field-collected *D. suzukii* flies were sent to the Systematic Entomology Laboratory, USDA, ARS, in Beltsville, MD, for taxonomic identification.

**Table 1:** Means (± SE) of *D. suzukii* captured in traps baited with different treatments in a preliminary field test conducted in Beltsville

Treatment	Replicates	Total no. SWD captured	Mean ± SE
Apple juice blend (7 components)*	3	845	281.67 ± 35.47*a*
Raspberry blend (11 components)**	3	230	76.67 ± 25.17*b*
2 MB	3	3	1.00 ± 1.00*c*
3 MB	3	9	3.00 ± 1.00*c*
2 MB + 3 MB	3	4	1.33 ± 0.58*c*
Blank control	3	1	0.33 ± 0.58*c*

Means in the same column followed by the different letters are significantly different at *α* = 0.05 (one-way ANOVA, Ryan–Einot–Gabriel–Welsch *F* test, *F* = 120.00, *df* = 5,12, *P *< 0.001). The numbers of other *Drosophila* spp. caught in this preliminary test were not counted. *Seven-component blend includes isobutanol (IB), 2-methyl butanol (2 MB), 3-methyl butanol (3 MB), ethyl hexanoate (EH), acetoin (AT), ethyl octanoate (EO), and methyl benzoate (MB) in natural ratios of 4:7:7:0.3:1:0.3:2 (v/v). **Abraham et al. (2015)

Lure dispenser tubes and trap drowning solutions with captured insects were collected and replaced weekly unless otherwise indicated. Flies with morphological characters (approximately 2–3.5 mm in length and 5–6.5 mm in wingspan with light yellow or brown body with red eyes, dark unbroken bands across the abdominal segments) were counted in the laboratory. *Drosophila suzukii* flies were identified with dissecting scopes based on the presence of a dark spot on the wing tips of males and the presence of the serrated ovipositor in females (Walsh et al. 2011). Number of male may not be accurate because young males (< 24 h) sometimes may lack the wing spot (Cini et al. 2012; Hauser 2011). Other *Drosophila* species collected in the traps were not identified and cited as “other *Drosophila* spp.” in 2015, 2016, and 2017 field tests. Because the blank control traps caught almost nothing, they were not included in the subsequent field tests unless otherwise indicated.

In 2015, three additional treatments, including treatment 14 (two components, including AT and EO in 1:1 ratio), treatment 16 (single component, AT), and treatment 17 (single component, EO), were placed in cotton balls in centrifuge tubes. The rest of compounds, including EtOH (10 mL) and AA (1 mL), were added into drowning water and evaluated as individual attractant or synergistic attractive agent when ever needed (Fig. 4, *N* = 3–15). In 2016, ACV (300 mL, Essential Everyday^®^, 5% acidity, Shoppers Food and Pharmacy, College Park, MD) and a commercially available Scentry^®^ SWD lure (Great Lakes IPM Inc., Vestaburg, MI) were used as standard lures for SWD attraction activity comparison. In addition, the amount of AA in the drowning solution in each trap was increased from 1 to 15 mL to reach the same acidity (5%) with ACV. In addition, ethyl acetate (EA) (5 mL) and phenethyl alcohol (PE) (1 mL) were added to the drowning solution and evaluated as individual attractants or synergistic attractive agents in 2016 (Fig. 5a–c, *N* = 6). Furthermore, additional tests using quinary blend (AT + EO + EA + AA + PE) were conducted at the blueberry field in Butler’s Orchard, MD (Fig. 5d, *N* = 9).

In 2017, the controlled release rate dispenser of quinary blend (AT + EO + EA + AA + PE) was formulated by ChemTica International, S.A. (Heredia Province, Santa Rosa, Costa Rica) and compared with ACV, Sentry lure, and laboratory-made quinary blend formulation (in polypropylene flex micro-centrifuge tube) at the blueberry field during the middle of blueberry field season (June 29–July 5 at the Butler’s Orchard, MD (Table 3, *N* = 12).

### Laboratory behavioral bioassay

Given that the apple juice seven-component blend **(**treatment 1) was attractive to *D. suzukii* in the preliminary field tests conducted in 2014 (Table 1), a laboratory dual-choice behavioral bioassay was designed to refine this blend in order to identify the key attractive component and examine the other factors, which may have influence to SWD attraction. A complete test apparatus consisted of a plastic cup containing two trap tubes with two lure vials, and a water wick (Fig. 2). The plastic cups were clear, cylindrical polypropylene food containers (946 mL, diameter 114 mm, height 127 mm, Paper Mart, CA, USA). The lid of each cup had an 80-mm-diameter circular hole cut in it, which was covered with a nylon mesh (no-thrips insect screen, mesh size: 81 × 81, Bioquip, CA, USA) to provide ventilation while retaining flies. The polystyrene trap tubes (95 mm × 28.5 mm, same as those used in insect rearing) were labeled “T” for treatment and “B” for blank control, respectively, and were then given the appropriate lure. Lures were either tested chemical/chemical blends (unless otherwise indicated, 20 µl pure individual or blend) loaded onto a small cotton ball held within a small polyethylene lure vial (26 mm × 8 mm × 1.5 mm thickness, Just Plastic Ltd., Norwich, UK) or blank controls of plain cotton or cotton with solvent (in dose response trials) held in the same type of small lure vial. Each lure vial (open lid) was placed vertically within its trap tube, and then, the top of each trap tube was sealed with Parafilm^®^ (Pechiney Plastics Packaging Inc., Menasha, WI, USA) leaving only a 4-mm-diameter hole in the center to provide an entrance for attracted flies. Each pair of loaded trap tubes was then placed vertically on opposite sides of the test cups with the entrance holes facing upwards. A water wick consisting of a glass scintillation vial (20 mL, VWR, PA, USA) filled with deionized water and plugged with a cotton ball was laid on the bottom of the plastic cup to serve as a water source for the flies during the experiment (Fig. 2). Flies were immobilized using a CO_2_ stream, and ten were transferred into each testing apparatus. The fully assembled test apparatus cups were then covered with their ventilated lids.

**Fig. 3:**
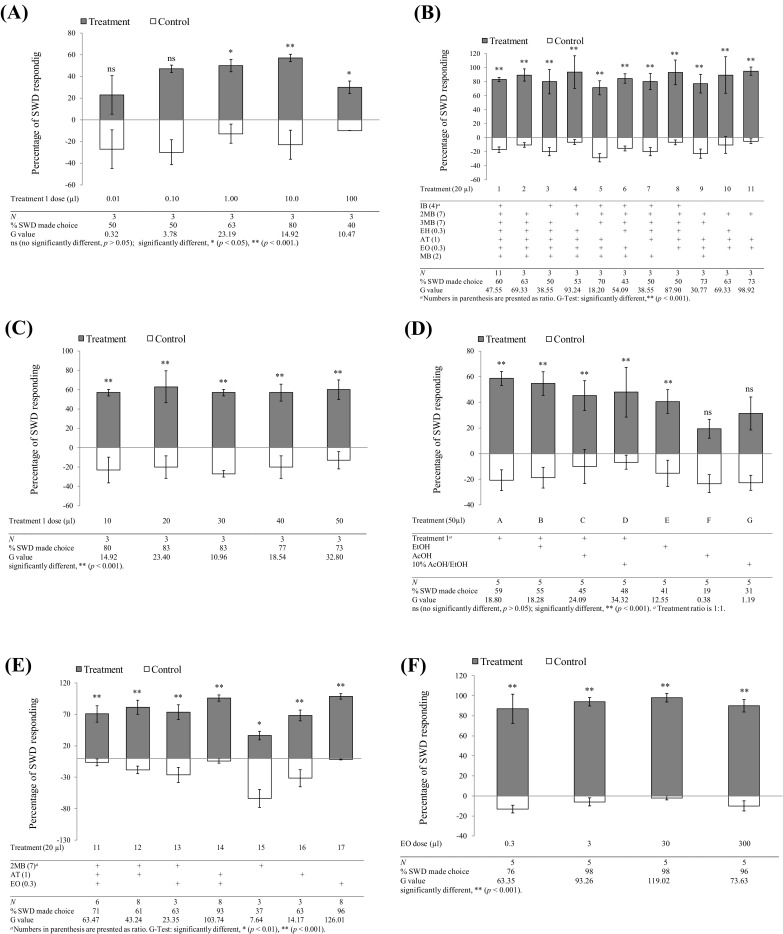
Behavioral responses (mean ± SE) of adult *D. suzukii* (male and female) against blank control in laboratory dual-choice bioassay to: **a** treatment 1 (seven components, 0.01–100 µl dose response); **c** treatment 1 (seven components, 10–50 µl dose response); **d** treatment 1 (seven components, effects of ethanol, acetic acid, and 10% acetic acid/ethanol solution); **b** treatment 1 (seven components), 2–8 (six components), 9 (five components), and 10 (four components), and 11 (three components); **e** treatments 11 (three components), 12, 13, 14 (two components), and 15, 16, 17 (single component); **f** treatment 17 (single component, EO, 0.3–300 µl dose response). Data are average percent of flies choosing one of the tubes 48 h after release. Experiments were replicated 3–11 times with ten flies each. Trapping data were analyzed by *G* tests: ns (not significantly different, *P *> 0.05), significantly different, *P < 0.05, **P < 0.001

In order to determine the optimized amount of chemical to be used in dual-choice laboratory experiments, a dose response experiment (treatment 1 vs. blank control) was first carried out (*N* = 3, Fig. 3a, c). Since ethanol (EtOH) and acetic acid (AA) have been previously reported as the major SWD attractants (Cha et al. 2014), they were examined individually and in different combinations with treatment 1 (50 µl total volume, *N* = 5, Fig. 3d). Component exclusion test was then performed to identify the key attractive components (*N* = 3–11, Fig. 3b). Individual and different combinations of three final attractive candidates were evaluated (*N* = 3–8, Fig. 3e). Finally, a dose response test of the most attractive component was assessed (*N* = 5, Fig. 3f). All assays were conducted in a fume hood (120 cm length × 60 cm width × 70 cm height) using new lures and young flies (*N* = 3–11, 2–8 days post-eclosion, directly from cornmeal diet rearing tubes with unknown mating status) under 25 °C, 60% RH, and a 16:8 h (L/D) photoperiod. Mixed-sex *D. suzukii* were used in bioassays unless stated otherwise, and all flies within and outside treatment and control trap tubes were counted and sexed after 48 h.

### Lure release rate

Release rates of three different kinds of attractants, including acetoin (AT), ethyl octanoate (EO), and a blend of AT and EO (ratio = 1:1), were measured under controlled conditions in a laboratory fume hood. Each attractant (1 mL) was loaded onto cotton balls held in open micro-centrifuge tubes (1.5 mL, VWR International, Radnor, PA). Fifteen of these micro-centrifuge tubes (*N* = 5) were suspended on hooks in a fume hood (temperature 20–25 °C, face velocity 129 feet/min). Each tube was weighed using an Ohaus GA110 analytical electronic balance **(**Pine Brook, NJ) every 24 or 72 h (weekend), and the amount of attractant residue was calculated and recorded over a period of 2 weeks.

### Statistical analyses

Data from bioassays that evaluated the SWD response to treatment trap tubes versus control trap tubes in dual-choice tests were converted to percentages and analyzed using *G* tests (Microsoft Office Excel 2007) (Sokal and Rohlf 1995) with the null hypothesis that *D. suzukii* would be in a 1:1 distribution (50% of choices of each trap tube). The mean number of *D. suzukii* in different treatment trap tubes for laboratory assays (captures/2 days) and that in different treatment traps (captures/week) in field trials were compared by one-way analysis of variance (ANOVA) followed by Ryan–Einot–Gabriel–Welsch *F* test (SPSS 10.0 for Windows) (George and Mallery 2002). Some field data ranges showed a strong positively skewed distribution (skewness/standard error of skewness > 1.96; therefore, the null hypothesis of normality was rejected); therefore, square root transformations were performed to remedy non-normality prior to statistical analyses. All statistical comparisons were considered for significance at *α* = 0.05. Attractants release rate data (decline of different kinds of attractants from open micro-centrifuge tubes) were fitted with exponential trendline (Microsoft Office Excel 2007), from which the half-life times (*t*_½_) of the lures were calculated.

## Results

### Identification of the volatile compounds from apple juices

GC–MS analyses of apple juice headspace extracts revealed that several volatile compounds were present in comparable amounts in both fresh and fermented samples (Fig. 1). Since it has been reported that the yeast microbe volatiles could mediate *Drosophila* flies attraction (Becher et al. 2010, 2012), special attention was given to the compounds produced and/or enriched during the fermentation process. We found that consistently higher levels (> 10× fold ratio relative to fresh apple juice volatile extract) of five compounds were associated with fermented apple juice volatile extract (Fig. 1, top trace). The compounds were identified as isobutanol (IB, peak 2), 2- and 3-methyl butanol (2 and 3 MB, peak 6), 3-hydroxy-2-butanone ([also called as acetoin (AT)], peak 10), and acetic acid (AA, peak 15) in an approximate ratio of 4:7:7:1:8 (v/v), respectively. Four compounds, including ethyl hexanoate (EH, peak 8), ethyl octanoate (EO, peak 14), ethyl decanoate (ED, peak 18), and methyl benzoate (MB, peak 19), were only produced by the fermentation in an approximate ratio of 0.3:0.3:0.2:2 (v/v), respectively, and absent in fresh apple juice volatile sample (Fig. 1, bottom trace). Compound, phenethyl alcohol (PE, peak 20), was also a volatile component enriched by fermentation. Two additional compounds, ethyl acetate (EA) and ethanol (EtOH), were detected as major headspace volatile components by SPME sampling method from both fresh and fermented apple juices. They were masked by solvent peak in conventional GC–MS analyses of apple juice headspace extracts.

### Preliminary field test

During a 1-week preliminary field test from October 17–24, 2014, at Beltsville, MD, a total of 845 adult *D*. *suzukii* were captured in all traps baited with apple juice seven-component blend (treatment 1), 230 *D*. *suzukii* were trapped in all traps baited with raspberry 11-component blend (Abraham et al. 2015), and only 17 *D*. *suzukii* were caught in all traps baited with 2, 3, and 2 MB + 3 MB blend (the most abundant components in fermented apple juice) and blank control traps. The numbers of other *Drosophila* spp. caught in traps were not counted at this time. Our data demonstrated that traps baited with treatment 1 captured significantly more male and female *D*. *suzukii* than traps baited with other treatments and blank controls (*F* = 120.00; *df* = 5,12; *P *< 0.001) (Table 1), indicating that the apple juice seven-component blend (treatment 1) contained some critical attractive components for attracting *D. suzukii*.

### Laboratory dual-choice bioassays

#### Dose response of apple juice seven-component blend

The doses above 1 µl (~ 1 mg) were found to be attractive (0.01, 0.1, 1, 10, and 100 µl) when treatment 1 blend was tested (0.01: *G* = 0.32, *df* = 1, *P* = 0.57; 0.1: *G* = 0.3.78, *df* = 1, *P* = 0.052; 1: *G* = 0.23.19, *df* = 1, *P* = 1.47E−6; 10: *G* = 14.92, *df* = 1, *P* = 1.2E−4; 100: *G* = 10.47, *df* = 1, *P* = 0.012) (Fig. 3a). An additional dose response experiment, using 10, 20, 30, 40, and 50 µl, was conducted. Similar activities were obtained and no significant differences were found in this dose range (10: *G* = 14.92, *df* = 1, *P* = 1.12E−04; 20: *G* = 23.40, *df* = 1, *P* = 1.32E−06; 30: *G* = 10.96, *df* = 1, *P* = 9.34E−04; 40: *G* = 18.54, *df* = 1, *P* = 1.7E−05; 50: *G* = 32.80, *df* = 1, *P* = 1.02E−08) (Fig. 3c). Therefore, unless otherwise indicated, an amount of 20 µl (~ 20 mg) for each chemical or blend was used as the standard dose in all other experiments. Because the sex ratios of *D. suzukii* were found to be close to 1:1 in the entire trap and control tubes, sex ratio determination was omitted in subsequent laboratory experiments.

#### Effects of ethanol and acetic acid to apple juice seven-component blend

Our results indicated that treatment 1 (**A**, seven components) and EtOH alone (**E**), as well as combinations of treatment 1 with EtOH (**B**), AA (**C**), 10% AA in EtOH (**D**), were significantly more attractive compared to a blank control (A: *G* = 18.80, *df* = 1, *P* = 1.50E−05; B: *G* = 18.28, *df* = 1, *P* = 1.90E−05; C: *G* = 24.09, *df* = 1, *P* = 9.19E−07; D: *G* = 34.32, *df* = 1, *P* = 4.68E−09; E: *G* = 12.55, *df* = 1, *P* = 3.97E−04; F: *G* = 0.38, *P* = 0.54; G: *G* = 1.19, *df* = 1, *P* = 0.28) (Fig. 3c), while AA (**F**) and 10% AA/EtOH (**G**) alone were not attractive, and no synergistic effects were observed when treatment 1 blend was combined with EtOH (**B**), AA (**C**), and 10% AA/EtOH (**D**) treatments (Fig. 3d).

#### Determination of key components from the apple juice seven-component blend

To determine the key attractive components from the seven-component synthetic blend (treatment 1), seven six-component blends (treatments 2–8) were prepared by eliminating one component from treatment 1 and one five-, four-, and three-component (treatments 9–11) blends were prepared by eliminating two, three, and four components, respectively, and compared to treatment 1 (1: *G* = 47.55, *df* = 1, *P* = 5.64E−12; 2: *G* = 69.33, *df* = 1, *P* = 8.34E−17; 3: *G* = 38.55, *df* = 1, *P* = 5.34E−10; 4: *G* = 93.24, *df* = 1, *P* = 4.76E−22; 5: *G* = 18.20, *df* = 1, *P* = 2.00E−05; 6: *G* = 54.09, *P* = 1.92E−13; 7: *G* = 38.55, *df* = 1, *P* = 5.34E−10; 8: *G* = 87.90, *P* = 6.88E−21; 9: *G* = 30.77, *df* = 1, *P* = 2.90E−08; 10: *G* = 63.93, *df* = 1, *P* = 8.34E−17; 11: *G* = 98.92, *df* = 1, *P* = 2.62E−23) (Fig. 3b). Although no significant activity reduction was observed in this component exclusion experiment, the three-component blend (treatment 11) showed the same attractive capacity as the complete treatment 1 blend, indicating that this blend (2 MB, AT, and EO) may contain the key attractive components (Fig. 3b). Consequently, this three-component blend (treatment 11) was further tested as two-component blends and as individual components. The results clearly demonstrated that an individual component, 2 MB (treatment 15), exhibited repellent effect. When 2 MB was used alone, blank control tubes trapped significantly more *D*. *suzukii* than the 2 MB treatment tubes (treatment 15, Fig. 3e). In addition, 2 MB elicited the lowest percentage response from *D*. *suzukii*. Compared with the attractant component ethyl benzoate (EO, treatment 17) in which 96% of *D*. *suzukii* made choice, only 37% of *D*. *suzukii* made choice when 2 MB was present (treatment 15, Fig. 3e). However, the treatments 16 (AT) and 17 (EO) individually elicited significant attraction to *D*. *suzukii* and the EO elicited the higher percentage response (96%) than that of AT (63%) from *D*. *suzukii* (11: *G* = 63.47, *df* = 1, *P* = 1.63E−15; 12: *G* = 43.24, *df* = 1, *P* = 4.85E−11; 13: *G* = 23.35, *df* = 1, *P* = 1.35E−06; 14: *G* = 103.74, *df* = 1, *P* = 2.31E−24; 16: *G* = 14.17, *df* = 1, *P* = 1.70E−03; 17: *G* = 126.01, *df* = 1, *P* = 2.98E−29) (Fig. 3e). Different doses of EO (from 0.3 to 300 µl levels) were examined to determine whether the amount of this compound loaded on the bait might affect the biological activity. Significantly attractive activities were observed for all doses tested in this assay, even at the lowest dose 0.3 µl (0.3 mg) (0.3: *G* = 63.47, *df* = 1, *P* = 4.77E−15; 3: *G* = 93.26, *df* = 1, *P* = 4.64E−22; 30: *G* = 119.02, *df* = 1, *P* = 1.04E−27; 300: *G* = 73.63, *df* = 1, *P* = 9.50E−18) (Fig. 3f).

#### Activity comparison of ethyl octanoate with raspberry extract

Effectiveness of treatment 17 (EO, single component) was compared with treatment 1 (seven-component blend), treatment 11 (three-component blend), treatment 14 (two-component blend), and raspberry extract (50 µl loading). Trap tubes baited with the single component, EO, captured significantly more *D*. *suzukii* than trap tubes baited with treatment 1 (seven components) and treatment 11 (three components). In addition, EO (treatment 17) was also as attractive as treatment 14 (two components, composed of EO and AT) and natural raspberry extract (*F* = 10.167, *df* = 4,20; *P* < 0.001) (Table 2).

**Table 2:** Means (± SE) of *D. suzukii* captured in tubes baited with different treatments against blank control in dual-choice laboratory bioassays

Treatment	Replicates	Total no. tested	Total no. captured	Mean ± SE
1 (7 components)*	5	50	23	4.60 ± 2.19*c*
11 (2 MB + AT + EO)	5	50	26	5.20 ± 2.28*bc*
14 (AT + EO)	5	50	47	9.40 ± 0.55*a*
17 (EO)	5	50	49	9.80 ± 0.45*a*
Raspberry extract	5	50	38	7.60 ± 1.81*ab*

Means in the same column followed by the different letters are significantly different at *α* = 0.05 (one-way ANOVA, Ryan–Einot–Gabriel–Welsch *F* test, *F* = 10.167, *df* = 4,20, *p *< 0.001). *Seven-component blend includes isobutanol (IB), 2-methyl butanol (2 MB), 3-methyl butanol (3 MB), ethyl hexanoate (EH), acetoin (AT), ethyl octanoate (EO), and methyl benzoate (MB) in natural ratios of 4:7:7:0.3:1:0.3:2 (v/v)

### Additional field tests

#### 2015

**Fig. 4:**
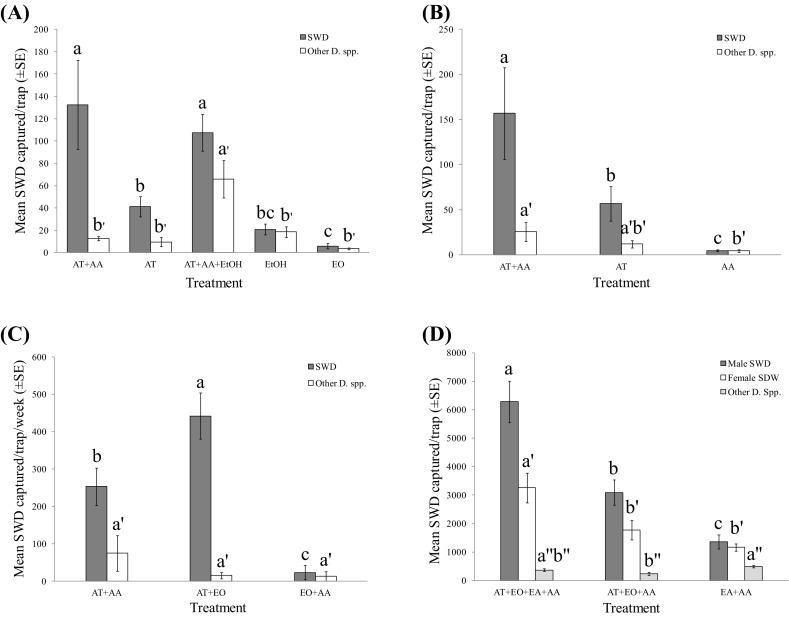
Mean (± SE) trap catches of *D. suzukii* and other *Drosophila* spp. in traps baited with different combinations of acetoin (AT), acetic acid (AA), ethyl octanoate (EO), ethanol (EtOH), and ethyl acetate (EA) deployed at the Beltsville Agricultural Center, Maryland. Identically colored bars with the different letters and superscripts above them are significantly different at *α* = 0.05 (one-way ANOVA, square root transformed, Ryan–Einot–Gabriel–Welsch *F* test). **a** Oct. 14–Oct. 21, 2015 (*N* = 10, *df* = 4,45; for SWD: *F* = 15.78; *P *< 0.0001; for other *Drosophila* spp.: *F* = 9.71, *P *< 0.0001); **b** Oct. 21–Oct. 28 (*N* = 15, *df* = 2,42; for SWD: *F* = 12.89, *P *< 0.0001; for other *Drosophila* spp.: *F* = 5.93, *P *< 0.01); **c** Oct. 28–Nov. 18, 2015 for 3 weeks (*N* = 3, *df* = 2,6; for SWD: *F* = 7.45, *P *< 0.05; for other *Drosophila* spp.: *F* = 1.49, *P *= 0.30); **d** Nov. 8–Nov. 15, 2016 (*N* = 3, *df* = 2,6; for male: *F* = 63.77, *P *< 0.0001; for female: *F* = 16.25, *P *< 0.01; for other *Drosophila* spp.: *F* = 7.06, *P *< 0.05)

Given that the single component, ethyl octanoate (EO, treatment 17), was the most attractive component for *D. suzukii* in laboratory bioassays (Fig. 3e, f), it was tested at Beltsville, Agricultural Research Center, MD, in the late fall during October 14 to November 18, 2015. Interestingly, EO alone did not show any activity at all, while the AT (treatment 16) was significantly more attractive than EO (*F* = 15.78, *df* = 4,45, *P *< 0.0001) (Fig. 4a). In addition, the attraction of AT was significantly enhanced when it was combined with (AA), but this enhanced effect was not observed for nontarget *Drosophila* species (for SWD: *F* = 15.78, *df* = 4,45, *P *< 0.0001; for other *Drosophila* spp.: *F* = 9.71, *df* = 4,45, *P *< 0.0001) (Fig. 4a). Furthermore, activity of EtOH was evaluated in the field. The same result as laboratory bioassay was obtained; EtOH alone did not show significant activity; instead, it attracted significantly more nontarget *Drosophila* when it was combined with the AT + AA blend (for other *Drosophila* spp., *F* = 9.71, *df* = 4,45, *P *< 0.0001) (Fig. 4a). The synergistic effect of AA to AT was confirmed in a later test. Significantly more *D. suzukii* were caught in the trap baited with AT when AA was added to the drowning solution than the traps baited with AT or AA alone (*F *= 12.89, *df* = 2,42; *P* < 0.001) (Fig. 4b). Although EO alone did not show any activity in the field (Fig. 4a), it significantly enhanced the attraction of AT (AT/EO = 1:1), but did not affect the trap catch of other *Drosophila* species (for SWD: *F* = 7.45, *df* = 2,6, *P *< 0.05; for other *Drosophila* spp.: *F* = 1.49, *df* = 2,6, *P *= 0.30) (Fig. 4c). No synergistic effect was observed when EO was combined with AA.

#### 2016

**Fig. 5:**
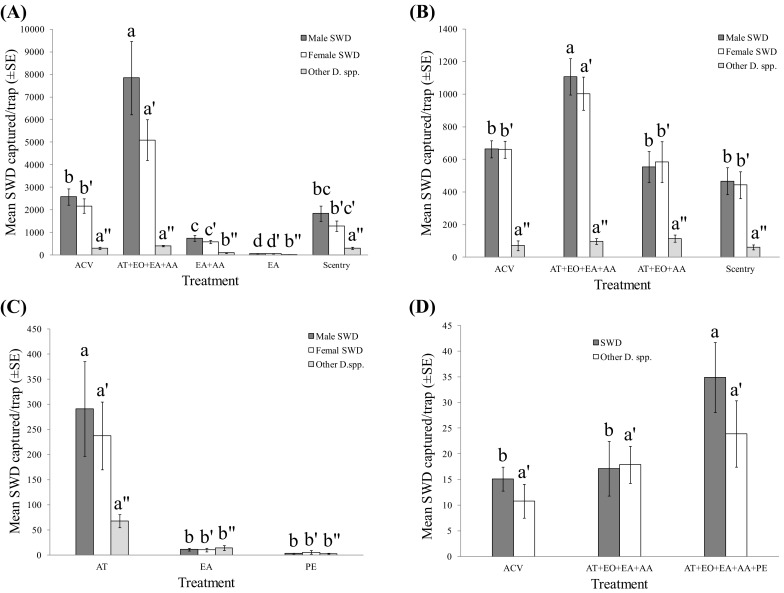
Mean (± SE) trap catches of *D. suzukii* and other *Drosophila* spp. in traps baited with different combinations of acetoin (AT), ethyl octanoate (EO), ethyl acetate (EA), acetic acid (AA), and phenethyl alcohol (PE), as well as apple cider vinegar (ACV) and commercial SWD lure from Scentry^®^ (Scentry) deployed at the Beltsville Agricultural Center and at the Butler’s Orchard blueberry field, Maryland. Identically colored bars with the different letters and superscripts above them are significantly different at *α* = 0.05 (one-way ANOVA, square root transformed, Ryan–Einot–Gabriel–Welsch *F* test). **a** November 10–18, 2016, at the Beltsville Agricultural Center (*N* = 6, *df* = 4,25; for male: *F* = 34.57, *P *< 0.0001; for female: *F* = 39.94, *P *< 0.0001; for other *Drosophila* spp.: *F* = 26.01, *P *< 0.0001); **b** November 25–December 22, 2016, for 27 days at the Beltsville Agricultural Center (total trap captures, lures, and contents were not changed and collected weekly) (*N* = 6, *df* = 3,20; for male: *F* = 10.38, *P *< 0.001; for female: *F* = 6.36, *P *< 0.01; for other *Drosophila* spp.: *F* = 1.12, *P *= 0.366); **c** December 2–22, 2016, for 20 days at the Beltsville Agricultural Center (total trap captures, lures, and contents were not changed and collected weekly) (*N* = 6, *df* = 2,15; for male: *F* = 9.09, *P *< 0.01; for female: *F* = 11.55, *P *< 0.001; for other *Drosophila* spp.: *F* = 18.61, *P *< 0.0001); **d** November 30–December 7, 2016, at the Butler’s Orchard blueberry field (*N* = 9, *df* = 2,24; for SWD: *F* = 4.47, *P *< 0.05; for other *Drosophila* spp.: *F* = 1.96, *P *= 0.163)

Our field data demonstrated that a mixture of EA and AA was moderately attractive to *D. suzukii*, but it was less attractive than a ternary blend (AT + EO + AA). However, when EA was added to this ternary blend to form a quaternary blend (AT + EO + EA + AA), attraction to *D. suzukii* was significantly increased, while nontarget *Drosophila* attraction was not (for male: *F* = 63.77, df = 2,6, *P *< 0.0001; for female: *F* = 16.25, *df* = 2,6, *P *< 0.01; for other *Drosophila* spp.: *F* = 7.06, *df* = 2,6, *P *< 0.05; for total SWD: *F* = 52.24, *df* = 2,6, *P *< 0.001) (Fig. 4d). The quaternary blend (AT + EO + EA + AA) was also significantly more attractive to *D. suzukii* than the widely used ACV and commercially available Scentry^®^ SWD lure under field conditions (for male: *F* = 34.57, *df* = 4,25, *P *< 0.0001; for female: *F* = 39.94, *df* = 4,25, *P *< 0.0001; for other *Drosophila* spp.: *F* = 26.01, *df* = 4,25, *P *< 0.0001; for total SWD: *F* = 37.16, *df* = 4,25, *P *< 0.0001) (Fig. 5a). However, like AA and EtOH, EA itself was not attractive in the field (Fig. 5a). The population of SWD was unusually high in the middle of November in Beltsville, MD. During a 1-week trapping period, a total of ~ 77,600 *D. suzukii* were captured by traps baited with the quaternary blend (AT + EO + EA + AA), yielding an average of 13,000 *D. suzukii* per trap. During the same period, a total of ~ 28,500 *D. suzukii* were captured by traps baited with ACV, yielding an average of 4700 *D. suzukii* per trap and a total of ~ 18,800 *D. suzukii* were captured by traps baited with Scentry^®^ lure, yielding an average of 3100 *D. suzukii* per trap. In addition, traps baited with the quaternary blend (AT + EO + EA + AA) captured much less nontarget *Drosophila* than ACV and Scentry^®^ lures, thus demonstrating its higher selectivity for *D*. *suzukii* attraction (SWD/other *Drosophila* spp. ratio: quaternary blend = 31.95, ACV = 15.90, Scentry = 10.16) (Fig. 5a). The synergistic effect of EA was further confirmed in a subsequent 4-week field test (total captures, lures, and contents were not changed weekly). The newly formed quaternary blend (AT + EO + EA + AA) attracted significantly more *D*. *suzukii* than the ternary blend (AT + EO + AA), ACV, and Scentry^®^ lures, but did not affect attraction of other *Drosophila* species (for male: *F* = 10.38, *df* = 3,20, *P *< 0.001; for female: *F* = 6.36, *df* = 3,20, *P *< 0.01; for other *Drosophila* spp.: *F* = 1.12, *df* = 3,20, *P *= 0.366; for total SWD: *F* = 8.86, *df* = 3,20, *P *< 0.001) (Fig. 5b). Again, the quaternary blend (AT + EO + EA + AA) demonstrated the highest SWD/other *Drosophila* spp. ratio during this period (quaternary blend = 11.43, ACV = 9.29, Scentry = 7.55).

Fermented apple juice also produced significantly more PE (compound **20**, Fig. 1) compared to fresh apple juice. When tested in the field, PE, like EA, did not show any activity for *D*. *suzukii* attraction compared to acetoin AT (for male: *F* = 9.09, *df* = 2,15, *P *< 0.01; for female: *F* = 11.55, *df* = 2,15, *P *< 0.001; for other *Drosophila* spp.: *F* = 18.61, *df* = 2,15, *P *< 0.0001) (Fig. 5c). However, by adding the PE into the quaternary blend (AT + EO + EA + AA) to form a quinary blend (AT + EO + EA + AA + PE), it significantly enhanced SWD attraction, but did not affect trap catch of nontarget *Drosophila* at the Butler’s Orchard blueberry field (for SWD: *F* = 4.47, *df* = 2,24, *P *< 0.05; for other *Drosophila* spp.: *F* = 1.96, *df* = 2,24, *P *= 0.163) (Fig. 5d).

#### 2017

Activity of a quinary blend (AT + EO + EA + AA + PE) was further confirmed at the Butler’s Orchard blueberry field during the middle of blueberry field season. ChemTica controlled release rate dispenser and our laboratory-made quinary blend formulation caught ~ 72 and ~ 47% *D*. *suzukii*, respectively, which were significantly more selective for *D*. *suzukii* attraction than Scentry lure (~ 27%) and ACV (~ 6%) formulations (for male SWD: *F* = 18.38, *df* = 4,55, *P *< 0.0001; for female SWD: *F* = 13.89, *df* = 4,55, *P *< 0.0001; for other *Drosophila* spp.: *F* = 27.27, *df* = 4,55, *P *< 0.0001; for other Diptera: *F* = 17.62, *df* = 4,55, *P *< 0.0001, for other arthropods: *F* = 1.60, *df* = 4,55, *P *= 0.19; for SWD%: *F* = 72.77, *df* = 4,55, *P *< 0.0001), although Scentry lure caught significantly more *D*. *suzukii* (Table 3).

**Table 3:** Means (± SE) of *D. suzukii* and other arthropods captured per trap at the Butler’s Orchard blueberry field

Treatment	♂ SWD	♀ SWD	Other *Drosophila* spp.	Other Diptera	Other arthropods	SWD %
Control	0*b*	0.08 ± 0.08*b*	0*b*	0.83 ± 0.21*b*	4.25 ± 0.69*a*	1.04 ± 1.04*d*
ACV	0.75 ± 0.35*b*	1.67 ± 0.41*b*	11.00 ± 1.82*b*	10.25 ± 2.04*a*	13.25 ± 3.69*a*	6.40 ± 1.62*d*
Scentry	15.25 ± 3.05*a*	41.00 ± 9.02*a*	116.08 ± 21.21*a*	7.00 ± 1.43*a*	21.17 ± 18.48*a*	26.84 ± 2.75*c*
ChemTica	4.67 ± 1.18*b*	18.08 ± 4.19*b*	2.75 ± 0.99*b*	0.17 ± 0.11*b*	3.00 ± 1.54*a*	72.30 ± 5.51*a*
Quinary blend	1.17 ± 0.36*b*	6.58 ± 1.98*b*	6.25 ± 1.13*b*	0*b*	1.67 ± 0.50*a*	47.24 ± 4.27*b*

Means in the same column followed by the different letters are significantly different at α = 0.05 (one-way ANOVA, Ryan–Einot–Gabriel–Welsch *F* test. *N *= 12, *df* = 4,55). For male SWD: *F* = 18.38, *P *< 0.0001; for female SWD: *F* = 13.89, *P *< 0.0001; for other *Drosophila* spp.: *F* = 27.27, *P *< 0.0001; for other Diptera, *F* = 17.62; *P *< 0.0001; for other arthropods, *F* = 1.60; *P *= 0.19; for SWD%: *F* = 72.77, *P *< 0.0001. No pesticide sprayed during trapping experiment

### Release rates of major attractants

**Fig. 6:**
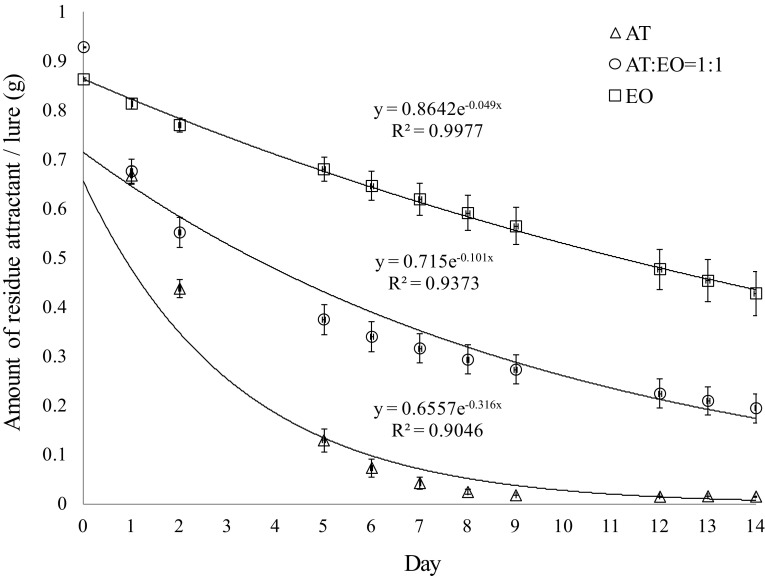
Mean (± SE) residual amounts of acetoin (AT), ethyl octanoate (EO), and blend of AT and EO (ratio = 1:1) measured in micro-centrifuge tube dispensers after exposure under fume hood conditions for a period of 2 weeks

Our laboratory release rate study demonstrated that the major attractant AT, close-range attractant EO, and a blend of AT and EO (ratio = 1:1) were desorbed from micro-centrifuge tube dispensers following first-order kinetics (Fig. 6, for AT, *r*^2^ = 0.9046; for blend of AT and EO, *r*^2^ = 0.9373; for EO, *r*^2^ = 0.9977). The decreases in volatile ingredients over time were best described by the following equations: for AT, *Y* = 0.6557e^−0.316t^, for blend of AT and EO, *Y* = 0.715e^−0.101t^, and for EO, *Y* = 0.8642e^−0.049t^. Half-life time of dispensers can be calculated by the equation: *t*_1/2_ = 0.693/*k*. Thus, micro-centrifuge tube dispensers with 1-mL loadings will release 50% of AT in ~ 2 days, EO in ~ 14 days, and AT and EO blend (ratio = 1:1) in ~ 7 days.

## Discussion

Chemical analyses of the headspace volatiles of apple juices using GC–MS revealed 20 identified aromas, of which some were increased in fermented apple juice and others were only produced by the fermentation. EO was a close-range attractant; it revealed the most powerful attracting capacity to *D. suzukii* in laboratory bioassays, but showed no activity by itself under field conditions. However, addition of EO into the binary blend of AT and AA to form a ternary blend (AT + EO + AA) significantly enhanced SWD attraction in the field. Ethanol, which has been previously reported as a principal component for SWD attraction from apple cider vinegar, wine, and yeast baits, did not enhance the activity of AT and AA. However, it did significantly decrease the SWD specificity of the blend (from ~ 90 to ~ 30%) by attracting many other nontarget insect species into the traps.

The compound, 3-hydroxy-2-butanone, also known as acetoin, is one of the volatile components found in increased amounts in fermented apple juice. It exists widely in nature and mainly used in baked foods as additive to enhance flavor (Bratovanova 2001; Ensminger et al. 1994; Xiao and Lu 2014a, b). It can be found in apples, butter, yogurt, asparagus, blackcurrants, blackberry, wheat, broccoli, brussels sprouts, and cantaloupe as food flavoring and fragrance (Aili et al. 2012; Bratovanova 1997; de Figueroa et al. 2001) and is an important physiological metabolite excreted by many microorganisms (Romano et al. 1993; Xiao and Xu 2007). Its threshold value in wine is very high, being about 150 mg/L (Romano and Suzzi 1996). AT has been identified from apple cider as a volatile component (Kahn et al. 1966; Rinaldi et al. 1974). A large amount of AT was produced by the vinegar fermentation produced, while only trace was released by the yeast fermentation of cider (Kahn et al. 1966; Rinaldi et al. 1974). It is similar to our result; a significant amount of AT was produced by the fermented apple juice, while only trace was released by the fresh apple juice (compound 10, Fig. 1). In addition, AT has also been shown as a semiochemical to attract insects (Said et al. 2005; Sreng 1990, 1993; Tolasch et al. 2003; Vlasakova et al. 2008; Witzgall et al. 2010). For SWD, it has been reported that a four-component synthetic bait [acetic acid, ethanol, acetoin, and methionol], in which the EtOH and AA were principal components for activity, was essential for SWD attraction (Cha et al. 2012, 2014, 2015). However, our data revealed that the single compound, AT, showed moderate activity in laboratory bioassays, but was a major semiochemical for SWD long-range attraction in the field.

The compound, ethyl octanoate, is also known as ethyl caprylate. It has been found in wine and produced during the fermentation process by yeast (Antonelli et al. 1999; Gallardo-Chacon et al. 2010; Patel and Shibamoto 2002; Tsakiris et al. 2010; Vianna and Ebeler 2001) and widely used in fragrances, flavorings, pharmaceuticals, and cosmetics. In addition, EO is known as one ingredient in multiple-component attractants for several insects including the fruit flies in the Tephritidae (El-Sayed 2017; Robacker et al. 1992; Toledo et al. 2009). However, to the best our knowledge, ethyl octanoate has not been reported as an attractant component for *D. suzukii*. Interestingly, although it exhibited the strongest activity for SWD attraction in the laboratory bioassay, it was not attractive to *D. suzukii* in field conditions, indicating that EO is a close-range attractant. It could significantly synergize the attraction of acetoin (AT) in the field.

Attractive activity of the ternary blend (AT + EO + AA) to *D. suzukii* was also significantly increased by adding ethyl acetate to form a quaternary blend (AT + EO + AA + EA). It was 2–4 times more attractive and 2–3 times more selective than the widely used ACV and commercially available SWD lures under field conditions, indicating that the EA is also a significant synergist for SWD attraction. Moreover, phenethyl alcohol, a volatile enriched during fermentation, also functioned as a strong synergistic agent. Our data indicated that a quinary chemical blend (AT + EO + AA + EA + PE) was more attractive than the quaternary blend (AT + EO + AA + EA) in field conditions and that neither EA nor PE was attractive to *D. suzukii* in the field.

Ethyl acetate is a major volatile component in plants (Malkina et al. 2011; Nonato et al. 2001) including many fruits (Krokida and Philippopoulos 2006; Nojima et al. 2003), as well as in wine (Ciani et al. 2006; Plata et al. 2003; Romano et al. 2003; Viana et al. 2008), beer (Jelen et al. 1998), whiskey (Carter and Linsky 1974), microbes (Lobs et al. 2016), and animal waste (Yasuhara 1987). It is an ester of ethanol and acetic acid and has been widely used as flavor enhancer in food and beverage production and as aroma enhancer in cosmetics and perfumes. It is affirmed by the United States Food and Drug Administration as GRAS (generally recognized as safe) (Opdyke 2013) and widely accepted as a safe food additive in many countries with E number E1504. In addition, EA is also widely used as a solvent for extracting organic compounds. Moreover, EA has also been reported as a component of pheromones or attractants for a variety of insects (El-Sayed 2017). For *D. suzukii*, our data clearly demonstrate that EA can significantly synergize the attraction of the ternary blend in the field; this is in contrast to previous findings that EA acts as a repellent that reduces the attraction of *D. suzukii* to blends of ethanol and acetic acid in both laboratory and field conditions (Cha et al. 2012).

The compound, phenethyl alcohol, occurs widely in nature. It is the main volatile component of rose aromas (Kim et al. 2010) and can also be found in many other essential oils, e.g., carnation, hyacinth, Aleppo pine, orange blossom, ylang-ylang, geranium, neroli, and champaca (Li et al. 2014). Because of its mild rose odor, PE has been extensively used in cosmetics, flavors, and perfumes (Scognamiglio et al. 2012). Interestingly, it also is an autoantibiotic produced by the fungus *Candida albicans* (Robin) Berkhout (Lingappa et al. 1969). As a common semiochemical, PE has been used by more than 90 different insect/arthropod species in their chemical communication (El-Sayed 2017), notably as a strong repellent against ticks (Thorsell et al. 2006). For *D. suzukii*, PE is one of the attractive components in the baits used by growers in the Pacific Coast states (Walsh et al. 2011). To the best our knowledge, PE has not been reported as single component to have any synergistic interactions with other components in *D. suzukii* attraction.

Most of the SWD baits published prior to our experiments were based on two major fermentation products, ethanol and acetic acid, and activity of these principal compounds could be synergized by several other chemicals (Cha et al. 2012, 2014, 2015; Kleiber et al. 2014; Landolt et al. 2012a, b). However, neither of these two compounds alone or in combination showed significant activities in our studies, although olfactory attraction of *D. suzukii* by symbiotic acetic acid bacteria has been observed in a two-way olfactometer bioassay (Mazzetto et al. 2016). Instead, acetic acid exhibited significantly synergistic effect to the SWD attraction of AT and ethanol had the undesirable effect of reducing lure specificity. In addition, the most common yeast bait and commercial lures had poor selectivity for *D. suzukii*. By comparing with yeast bait and several commercial available lures in cherry orchards, Kirkpatrick et al. (2017) found that the percentage of total captures in traps ranged from 31 to 39% for *D. suzukii* and 60 to 68% for nontarget flies. However, our quinary chemical blend (AT + EO + AA + EA + PE) showed much higher selectivity, which could trap 72% (ChemTica) and 47% (polypropylene micro-centrifuge tube formulation) *D. suzukii* in the field. This result also indicated that the controlled release rate dispenser had significantly higher activity and selectivity for *D. suzukii*.

Our laboratory release rate data indicated that EO not only functioned as a close-range attractant, but also could slow down the release rate of AT when they were blended together (*k*_AT_ = 0.316 < *k*_AT+EO_ = 0.101), thereby prolonging lure longevity. Considering that the average temperature in the field may be higher than in our laboratory, the actual rate constant (*k*) could be greater than that obtained in the laboratory. Therefore, the effective half-life for our baits may be shorter in the field than the calculated value. Our laboratory prepared dispensers baited with 1 mL AT and EO blend (ratio = 1:1) as the major SWD attractant in a quaternary blend (AT + EO + AA + EA) or a quinary blend (AT + EO + AA + EA + PE) could not be expected to provide more than 1 week of maximum SWD attraction under field conditions. Therefore, controlled release rate dispenser will resolve this problem.

Our identified SWD attractant blend based on fermentation may have significant impact on the management of this invasive species. As a demonstrated essential volatile component for SWD attraction, acetoin could be one of the major chemical cues used to attract *D. suzukii* to bait or monitoring stations. The newly identified quinary chemical blend (AT + EO + EA + AA, PE) will be examined for attract SWD at lower population levels and before extensive fruit injury can occur. In addition, elimination of EtOH as a bait component will increase lure specificity and allow traps to reduce nontarget captures of pollinators and other beneficial insects. Additional field tests will be conducted in orchards/fields during the growing season with the competitive presence of host fruits. The increased attractiveness and specificity of the quinary chemical blend may help accurately detect SWD adult infestations for timely pest management interventions, thereby reducing the need for conventional insecticide usage and ultimately protecting our environment and ecosystem.

## Authors’ contributions

All authors performed experiments. AZ and YF conceived the idea. AZ, YF, RB, and AR wrote the manuscript. All authors contributed to the discussion and reviewed the manuscript.

## Electronic supplementary material

Below is the link to the electronic supplementary material.

Supplementary material 1 (DOCX 2487 kb)

Supplementary material 2 (DOCX 2487 kb)

Supplementary material 3 (DOCX 3921 kb)

Supplementary material 4 (DOCX 6120 kb)
