# Supplementary figures and images for: Identification of attractive blend for spotted wing drosophila, Drosophila suzukii, from apple juice
Source: J Pest Sci (2004). 2018 Jun 22;91(4):1251–67. doi: 10.1007/s10340-018-1006-9 (PMC6063330; doi:10.1007/s10340-018-1006-9)

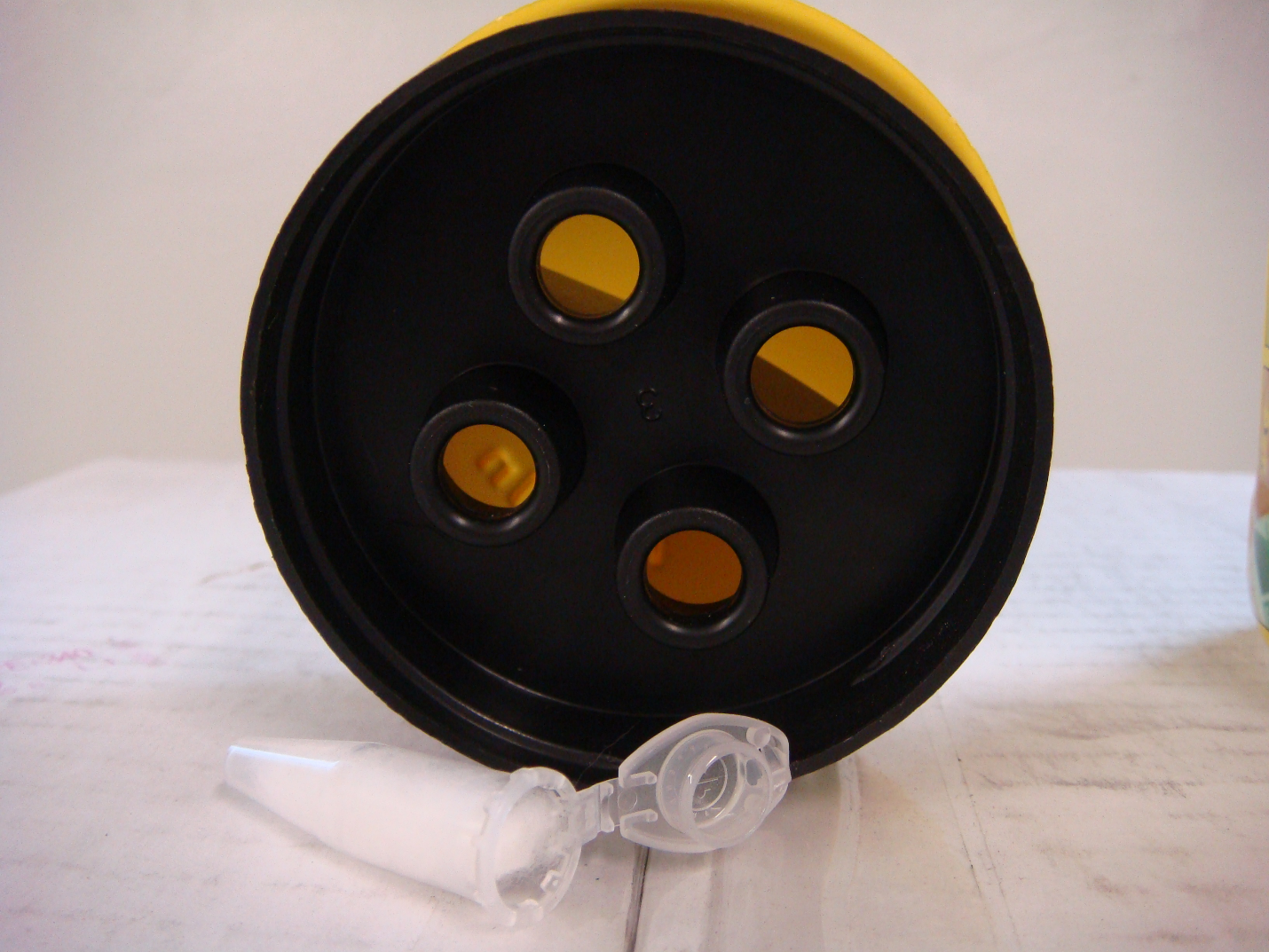


S1. Lure preparation

Supplement: Supplementary file 1 — Supplementary material 1 (DOCX 2487 kb) [file 10340_2018_1006_MOESM1_ESM.docx]

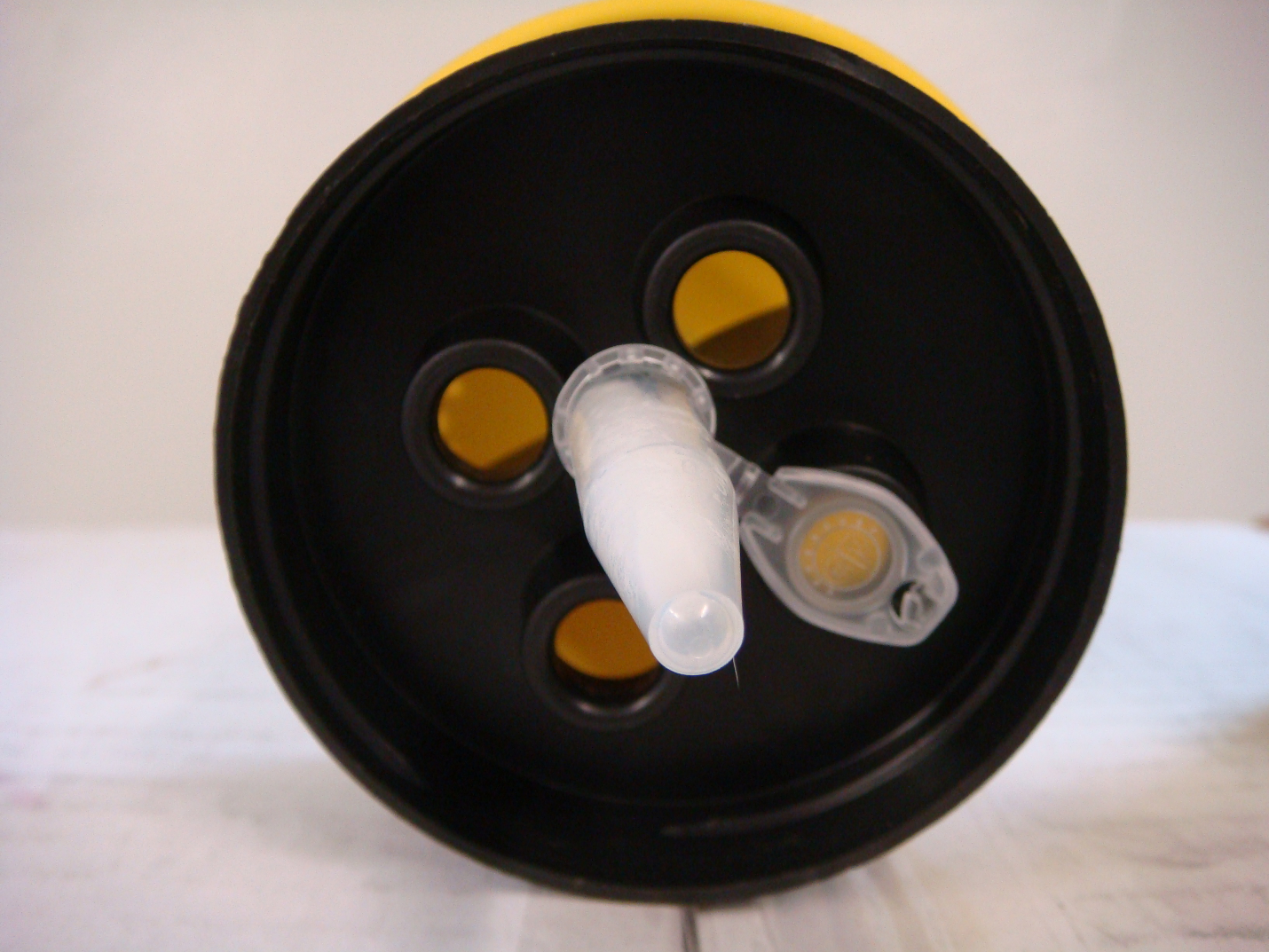


S2. Lure installation

Supplement: Supplementary file 2 — Supplementary material 2 (DOCX 2487 kb) [file 10340_2018_1006_MOESM2_ESM.docx]

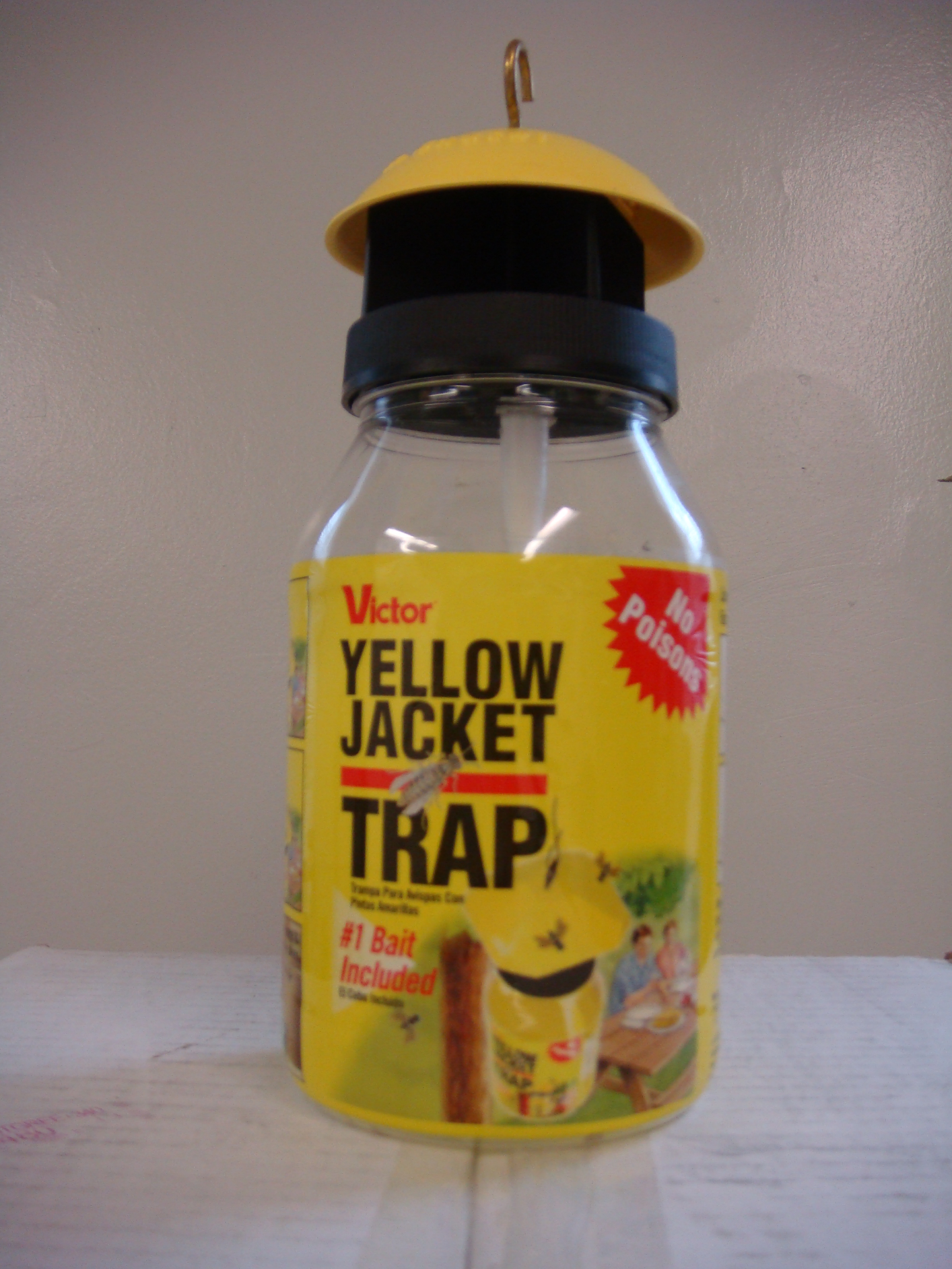


S3. Trap setting-up

Supplement: Supplementary file 3 — Supplementary material 3 (DOCX 3921 kb) [file 10340_2018_1006_MOESM3_ESM.docx]

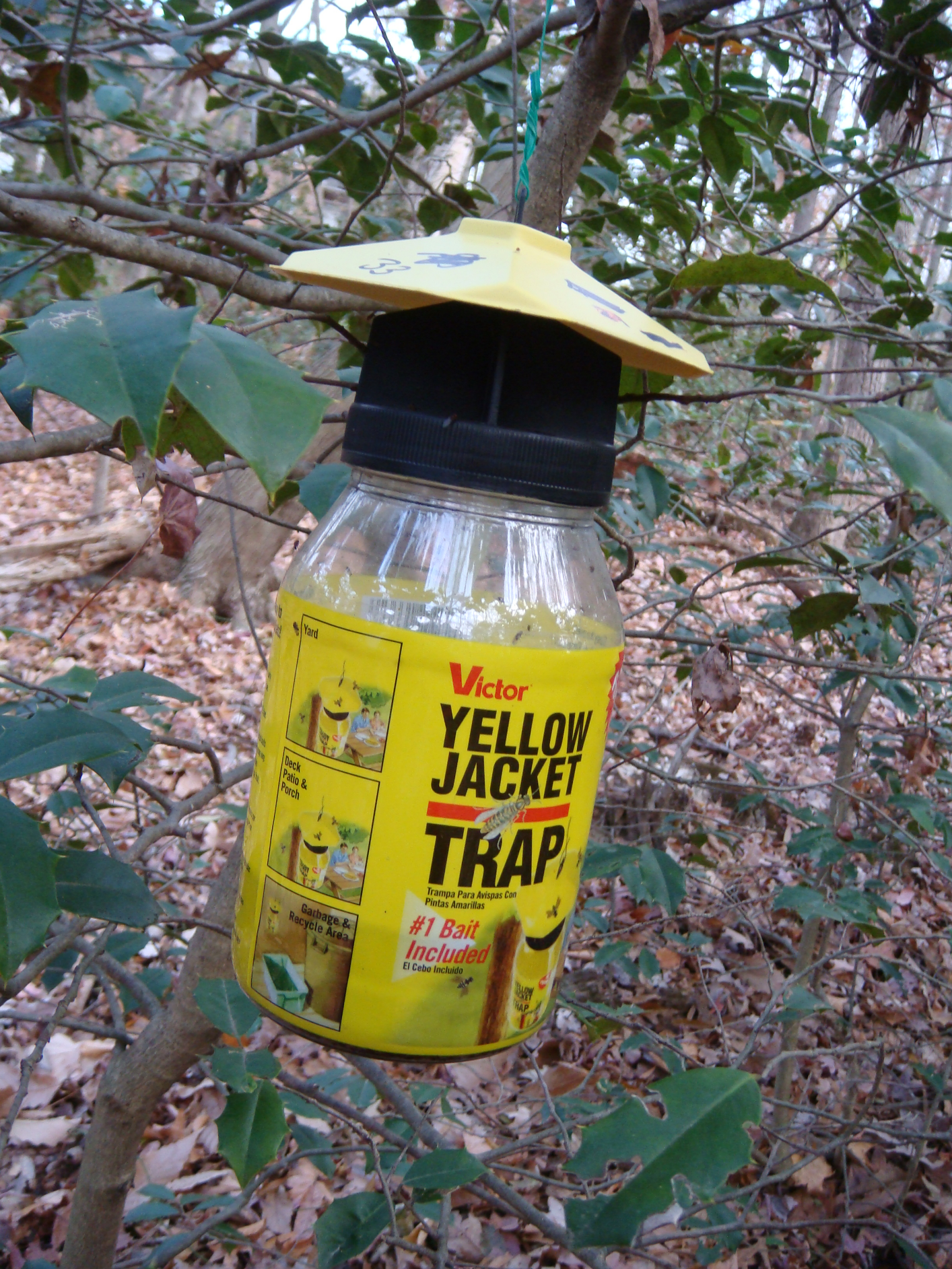


S4. Trap in the field

Supplement: Supplementary file 4 — Supplementary material 4 (DOCX 6120 kb) [file 10340_2018_1006_MOESM4_ESM.docx]
